# Supplementary material for: The evolution of human altriciality and brain development in comparative context
Source: Nat Ecol Evol. 2023 Dec 4;8(1):133–46. doi: 10.1038/s41559-023-02253-z (PMC10781642; doi:10.1038/s41559-023-02253-z)
Supplement: Supplementary file 1 — Reporting Summary [file 41559_2023_2253_MOESM1_ESM.pdf]

nature portfolio

Corresponding author(s): Aida Gomez-Robles

Last updated by author(s): Sep 27, 2023

Reporting Summary

Nature Portfolio wishes to improve the reproducibility of the work that we publish. This form provides structure for consistency and transparency in reporting. For further information on Nature Portfolio policies, see our Editorial Policies and the Editorial Policy Checklist.

Statistics

For all statistical analyses, confirm that the following items are present in the figure legend, table legend, main text, or Methods section.

n/a	Confirmed	
☐	☒	The exact sample size (*n*) for each experimental group/condition, given as a discrete number and unit of measurement
☐	☒	A statement on whether measurements were taken from distinct samples or whether the same sample was measured repeatedly
☐	☒	The statistical test(s) used AND whether they are one- or two-sided<br>*Only common tests should be described solely by name; describe more complex techniques in the Methods section.*
☐	☒	A description of all covariates tested
☐	☒	A description of any assumptions or corrections, such as tests of normality and adjustment for multiple comparisons
☐	☒	A full description of the statistical parameters including central tendency (e.g. means) or other basic estimates (e.g. regression coefficient) AND variation (e.g. standard deviation) or associated estimates of uncertainty (e.g. confidence intervals)
☐	☒	For null hypothesis testing, the test statistic (e.g. F, t, r) with confidence intervals, effect sizes, degrees of freedom and P value noted<br>*Give P values as exact values whenever suitable.*
☐	☒	For Bayesian analysis, information on the choice of priors and Markov chain Monte Carlo settings
☒	☐	For hierarchical and complex designs, identification of the appropriate level for tests and full reporting of outcomes
☐	☒	Estimates of effect sizes (e.g. Cohen's *d*, Pearson's *r*), indicating how they were calculated

Our web collection on statistics for biologists contains articles on many of the points above.

Software and code

Policy information about availability of computer code

Data collection	Data were compiled from the literature, and no specialized software was used.
Data analysis	Data analysis was carried out in R version 4.1.3 and BayesTraits V3 (available at http://www.evolution.rdg.ac.uk/BayesTraits.html). pANCOVA analyses were carried out using functions from the R package 'evomap', which is available at https://github.com/JeroenSmaers/evomap. Other standard packages for phylogenetic analysis (such as 'phytools', 'ape' and 'geiger') and plotting ('ggplot2', 'smplot2') were used.

For manuscripts utilizing custom algorithms or software that are central to the research but not yet described in published literature, software must be made available to editors and reviewers. We strongly encourage code deposition in a community repository (e.g. GitHub). See the Nature Portfolio guidelines for submitting code & software for further information.

Data

Policy information about availability of data

All manuscripts must include a data availability statement. This statement should provide the following information, where applicable:

- Accession codes, unique identifiers, or web links for publicly available datasets
- A description of any restrictions on data availability
- For clinical datasets or third party data, please ensure that the statement adheres to our policy

Datasets and scripts used to carry out this study are available through the link 10.6084/m9.figshare.22242724.

Human research participants

Policy information about studies involving human research participants and Sex and Gender in Research.

Reporting on sex and gender	N/A
Population characteristics	N/A
Recruitment	N/A
Ethics oversight	N/A

Note that full information on the approval of the study protocol must also be provided in the manuscript.

Field-specific reporting

Please select the one below that is the best fit for your research. If you are not sure, read the appropriate sections before making your selection.

☐ Life sciences ☐ Behavioural & social sciences ☒ Ecological, evolutionary & environmental sciences

For a reference copy of the document with all sections, see nature.com/documents/nr-reporting-summary-flat.pdf

Ecological, evolutionary & environmental sciences study design

All studies must disclose on these points even when the disclosure is negative.

Study description	The study includes and in-depth analysis of data on neonatal and adult brain and body size across 140 species of placental mammals. The study includes a comparison of the proportion of neonatal to adult brain and body size across the major orders of mammals. It also includes the measurement of branch-specific evolutionary rates across the mammalian phylogeny, as well as a comparison of the scaling relationships between neonatal and adult values between orders based on phylogenetic ANCOVAs. The final part of our study includes an inference of whether key neurodevelopmental events happened pre- or postnatally across different hominin species, which is based on published models by Workman et al (2013).
Research sample	The studied sample includes 140 species of placental mammals, for which data were obtained on neonatal brain size, adult brain size, neonatal body size, adult body size, gestation length, and generation time. Data were compiled from the literature using the sources referenced in the 'Methods' section of the manuscript. Data were obtained for all the species of placental mammals that we were able to find in the literature.
Sampling strategy	Sample size was not predetermined, but all species with available data were included in our study. Only one species-specific value was included for each species, as it was not possible to obtain multiple values for each species.
Data collection	Data were compiled by A.G.-R. and C.N. When available, tabulated data were obtained from the supplementary information files of relevant publications. Data from older papers were obtained from the paper and copied into our general compilation.
Timing and spatial scale	The initial compilation of data was carried out by A.G.-R. in 2016. In 2020, C.N. checked the initial dataset based on an independent literature review, and made amendments as necessary.
Data exclusions	Species were included in the study only if data were available for all the variables of interest (neonatal brain size, neonatal body size, adult brain size, and adult body size). In the few cases where we could not find data on gestation length or generation time for a given species, the missing value was replaced by that corresponding to the closest sister species within the same genus.
Reproducibility	We checked the reproducibility of our rate analyses by calculating ancestral values using a variable rates rjBM approach and a mvBM approach (function available in the R package 'evomap'), obtaining very similar results regarding human rates. We also repeated these analyses using the residuals of a PGLS regression between neonatal and adult values and comparing the measured rates with those obtained when measuring rates for the proportion of brain and body size at birth. We also recalculated evolutionary rates using a comparison of the observed amounts of change per branch with those obtained when simulating evolution over the mammalian phylogeny. Those rates show a very high correlation with the rates obtained by comparison of the observed amount of change per branch with the expected amount of change per branch based on branch lengths.
Randomization	The classification of mammalian species within the four major orders we have used in our study is well established and randomization is not relevant.
Blinding	The values used in our study are associated with the species they belong to, and they were reviewed based on this species identity. Therefore, blinding was not possible.

Did the study involve field work? ☐ Yes ☒ No

Reporting for specific materials, systems and methods

We require information from authors about some types of materials, experimental systems and methods used in many studies. Here, indicate whether each material, system or method listed is relevant to your study. If you are not sure if a list item applies to your research, read the appropriate section before selecting a response.

Materials & experimental systems

n/a	Involved in the study
☒	☐ Antibodies
☒	☐ Eukaryotic cell lines
☒	☐ Palaeontology and archaeology
☒	☐ Animals and other organisms
☒	☐ Clinical data
☒	☐ Dual use research of concern

Methods

n/a	Involved in the study
☒	☐ ChIP-seq
☒	☐ Flow cytometry
☒	☐ MRI-based neuroimaging
